# MiR-144-3p Targets FoxO1 to Reduce Its Regulation of Adiponectin and Promote Adipogenesis

**DOI:** 10.3389/fgene.2020.603144

**Published:** 2020-12-14

**Authors:** Weimin Lin, Yonghang Tang, Yuelei Zhao, Jindi Zhao, Lifan Zhang, Wei Wei, Jie Chen

**Affiliations:** College of Animal Science and Technology, Nanjing Agricultural University, Nanjing, China

**Keywords:** miRNAs, miR-144-3p, FoxO1, adiponectin, adipogenesis

## Abstract

MicroRNAs (miRNAs), as a series of important short-chain non-coding RNAs, play an important post-transcriptional role in many biological activities, including adipogenesis. miR-144 is significantly upregulated in type II diabetes (T2D), and is considered to be an important biomarker for T2D. However, although the occurrence of T2D is inextricably linked to adipogenesis, whether miR-144 directly regulates adipogenesis remains to be further explored. In this paper, we demonstrate that miR-144 has a higher expression level in a porcine high backfat group, and it has a significant positive effect on promoting the differentiation of pre-adipocytes. *FoxO1* is a target gene of miR-144, and inhibits the differentiation of pre-adipocytes. On the other hand, we demonstrate that FoxO1 can bind to the *AdipoQ* gene promoter, then regulate the *AdipoQ* expression by binding to the FoxO1 binding site in the *AdipoQ* promoter -1,499 to -1,489 bp and -1,238 to -1,228 bp regions, especially the -1,499 to -1,489 bp region. Meanwhile, miR-144 and FoxO1 co-expressional research has also shown that both factors regulate adipogenesis. To sum up, our research indicates that miR-144 targets *FoxO1*, thus reducing its expression and inhibiting its promotional effect on adiponectin, thereby alleviating the inhibitory effect of adiponectin on adipogenesis.

## Introduction

Obesity has gradually become one of the major threats to human health around the world. A batch of diseases such as type II diabetes (T2D), hypertension, and cardiovascular disease are closely related to being overweight or obesity (Kopelman, 2000; Goran et al., 2003). The adipose tissue developed can also trigger an immune mechanism which could create an inflammatory reaction (Hammarstedt et al., 2018). Adipocyte differentiation and proliferation lead to fat tissue expansion, and excessive fat tissue results in obesity-related metabolic syndromes. Hence, adipocytes are emerging as a significant target in the treatment of obesity-related metabolic syndromes in the clinical setting (Shen et al., 2018). In this context, it is very necessary to explore the molecular genetic mechanisms of adipocyte differentiation and proliferation.

Adipocyte differentiation and proliferation is controlled by an enormous and complicated interaction network, which involves hundreds of factors and genes. Among them, peroxisome proliferator activated receptor γ (PPARγ) has been confirmed to play a critical role in adipocyte development. As a downstream factor in the adipogenesis regulation network, PPARγ is involved in regulating insulin sensitivity (Barroso et al., 1999; Doney et al., 2004; Monajemi et al., 2007). Other important adipogenic factors include the CCAAT/enhancer binding proteins (C/EBP) family, such as C/EBPs α, β, γ, δ, ε, and ζ, which can form both homodimers and heterodimers to bind the promoter region of genes containing the CCAAT sequence to regulate the expression level (Hamm et al., 2001; Yu et al., 2015). Dexamethasone (DEX) and isobutyl-methylxanthine (IBMX) can promote C/EBPδ and C/EBPβ at the early stage of adipogenesis, respectively. Then, both of them *trans*-activate PPARγ and C/EBPα. PPARγ and C/EBPα regulate the expression of each other and both play central roles in adipogenesis (Cao et al., 1991).

Besides, there are other factors that play a regulatory role in adipogenesis, including microRNAs. Generally, microRNA (miRNA) is a non-coding RNA with ∼21 nucleotides. It differs in coding genes, for its length is too short to code, thus miRNAs play a post-transcriptional role by binding the 3′ untranslated region (3′UTR) of coding genes, or binding non-coding genes, for instance, long-coding RNAs (lncRNAS), circle RNAs (circRNAs), pseudogenes, and so on (Hamilton and Baulcombe, 1999; Grishok et al., 2001; Ketting et al., 2001). It regulates a series of biological activities, including adipogenesis. For instance, miR-27 inhibits lipoprotein lipase (*LPL*) which suppresses adipocyte differentiation (Zhang et al., 2014). miR-34 acts as an inhibitor of beige or brown fat formation (Fu et al., 2014). miR-130 suppresses adipogenesis by binding the 3′UTR of *PPAR*γ to inhibit its expression (Pan et al., 2014). Nevertheless, miR-103 is one of the few miRNAs that has a positive effect on adipogenesis, it can promote 3T3-L1 cell differentiation by targeting *MEF2D* and activating the Akt/mTOR signal pathway (Li et al., 2015). Except for this miRNA, miR-17 (Han et al., 2017), miR-199 (Shi et al., 2014), miR-425 (Chen et al., 2017), and miR-7134 (Wang et al., 2017) all regulate adipogenesis.

miR-144 is highly upregulated in T2D and has the ability to impair insulin signaling and thus regulate adipogenesis, it has even been used as a biomarker of T2D (Karolina et al., 2011; Liang et al., 2018). However, whether miR-144 directly regulates adipogenesis is still controversial. There is a report that suggests that miR-144 targets *C/EBP*α and thus inhibits adipocyte differentiation (Itziar et al., 2017), but another report demonstrates that it can promote adipogenesis by targeting *Klf3* and *CtBP2* (Shen et al., 2018). Here, this paper tries to explore the molecular mechanism of miR-144 in adipogenesis regulation.

## Materials and Methods

### Experiment Animals

The animals used in this study were Erhualian piglets of 7 days old. All of them were from the Changzhou Erhualian Pig Production Cooperation (Changzhou, Jiangsu, China).

All animal experiments including the pre-adipocytes collected were approved by the Animal Ethics Committee of Nanjing Agricultural University.

### Cell Culture

Subcutaneous adipose tissue was isolated from the piglets and soaked in phosphate-buffered saline (PBS). Adipose tissue was cut with scissor into 1 mm^3^ pieces, and then digested by 1 mg/mL collagenase type I (Invitrogen, Carlsbad, CA, United States) in a 37°C, 50 rpm/min shaking bath for over 2 h. Digestion was stopped by adding 1.5 times the volume of Dulbecco’s modified Eagle’s medium/Ham’s F-12 (DMEM-F12) growth medium (10% fetal bovine serum + 1% penicillin-streptomycin). The digested tissue was filtrated by 200 μm nylon mesh to collect the solution containing pre-adipocytes. The solution was centrifuged twice at 1,000 rpm/min for 10 min to collect the pre-adipocytes. The supernatant was removed and 4 ml of growth medium was added to resuspend the pre-adipocytes. The pre-adipocytes were cultured in the growth medium at 37°C with 5% CO_2_. The medium was replaced every 2 days.

### Cell Transfection and Differentiation

Cells were cultured in 6- or 12-well plates until its density was of 85% confluence. Purpose plasmids or oligonucleotides were transfected to the cells using Lipofectamine 3000 (Invitrogen, Shanghai, China) following the protocol. All the purpose plasmids, siRNAs, and miRNA mimics and inhibitors are shown in Supplementary Table S1.

The adipogenic differentiation inducer medium (DIM) was used to stimulate the pre-adipocytes whose density was of 85% confluence for almost 4 days.

The pre-adipocytes were then induced for adipogenic differentiation using the DIM inducer, which comprised of 2.5 μM dexamethasone, 8.6 μM insulin, 0.1 mM 3-isobutyl-1 methylxanthine (IBMX), 1% penicillin-streptomycin, and 10% FBS in Dulbecco’s modified Eagle’s medium/Ham’s- High Glucose (DMEM-HG) (Sigma-Aldrich, Shanghai, China). After adipogenic differentiation induction for 4 days, the medium was then replaced with maintenance medium containing 8.9 μM insulin and 10% FBS-DMEM-HG until day 8. The above medium was replaced with fresh medium every 2 days.

### RNA Isolation, Library Preparation, and RT-PCR

Total RNA was isolated using the Trizol reagent (TaKaRa, Dalian, China). The mRNAs and miRNAs cDNA libraries were reverse-transcribed by the PrimeScript^TM^ RT Master Mix (TaKaRa, Dalian, China) and the miRNA 1st Strand cDNA Synthesis Kit (by stem-loop) (Vazyme, Nanjing, China), respectively. Quantitative real time PCR (q-PCR) was performed using the AceQ Universal SYBR qPCR Master Mix (Vazyme, Nanjing, China) and the miRNA Universal SYBR qPCR Master Mix (Vazyme, Nanjing, China), respectively. The relative level of RNA expression was normalized to *GAPDH* and *U6* expression levels using the 2^–ΔΔCt^ method. Every sample was performed in triplicate. All the primers used are shown in Supplementary Table S1.

### Oil Red O Staining and Triglyceride Assay

Briefly, the differentiated porcine pre-adipocytes were gently washed three times with fresh 1×PBS and then fixed in 4% paraformaldehyde for 30 min. The fixed cells were washed three times with 1×PBS and stained with 60% saturated oil red O for 30 min (Sigma-Aldrich, Shanghai, China). Subsequently, the fixed cells were washed three times with 1×PBS. Images of the cells were captured using a Zeiss Axiovert 40 CFL inverted microscope (Thornwood, NY, United States).

Total triglyceride was quantified by the elution of oil red O with isopropanol and the absorbance was measured at the 510 nm wavelength.

### Luciferase Reporter Assay

The porcine pre-adipocytes were used as luciferase reporter vectors. The cells were cultured in 12-well plates until they reached 85% confluence. pGL3 vector containing the AdipoQ promoter region and FoxO1-CDs, pmirGLO vector containing the miR-144 binding region of FoxO1 3′UTR, and miR-144 mimic/NC or miR-144 inhibitor/NC were, respectively, co-transfected into the pre-adipocytes using Lipofectamine 3000 (Invitrogen Shanghai, China). Firefly and Renilla luciferase activity were quantified using the Dual-Luciferase Reporter Assay System (Promega, Madison, WI, United States).

### Chromatin Immunoprecipitation Assay (ChIP)

ChIP experiments used the ChIP Assay Kit (Boytime, Nanjing, China) following the manufacturer’s instruction. In brief, porcine pre-adipocytes were cross-linked with 1% formaldehyde at 37°C for 10 min. Cross-linking was quenched with 1× glycine for 5 min at RT. The ultrasonic cell smash machine VCX750 (Sonics, United States) was used to smash the cells and to obtain DNA fragments between 200 and 1,000 bp as verified by ethidium bromide electrophoresis. For immunoprecipitation, every 100 μl cell lysis buffer was incubated with 1 μg of antibody against FoxO1 (cat 383312 ZENBIO) at 4°C overnight. Immunoprecipitated complexes were isolated using 60 μl Protein A Agarose/SalmonSperm DNA at 4°C for 2 h, and were then washed with the following: low salt wash buffer, high salt wash buffer, LiCl wash buffer once, and TE buffer twice. Final ChIP DNA was subjected to PCR analysis using a *AdipoQ* promoter specific primer pair, as shown in Supplementary Table S1.

### Western Blotting

In brief, porcine pre-adipocytes were lysed and the total protein was extracted using radioimmunoprecipitation assay (RIPA) lysis buffer (Beyotime, Jiangsu, China), following the manufacturer’s instructions. A BCA Protein Assay kit (Beyotime, Jiangsu, China) was used to quantify the total extracted protein. Each protein sample (20 μg/well) was loaded into a 12% SDS-PAGE gel (Zoman, Beijing, China). Then the SDS-PAGE was transferred to a PVDF membrane (Millipore, Billerica, MA, United States) after 70 min of electrophoresis. Subsequently, the transferred membranes were blocked by TBST containing 5% non-fat dried milk for 2 h at 37°C, followed by overnight primary antibody incubation (ZenBioScience, Chengdu, China) at 4°C. Then, the immunoblot membranes were washed three times with 1×TBST for 15 min, and were incubated in horseradish peroxidase-conjugated secondary antibody for 2 h at 37°C. The blots were developed using the ECL Chemiluminescence Detection Kit (Vazyme, Nanjing, China), and were photographed using the VersaDoc 4000 MP system (Bio-Rad).

### Bioinformatics Analysis

Four kinds of online software were used to predict the miR-144-3p target genes, including Targetscan^1^, PicTar^2^, miRmap^3^, and miRanda^4^. The precursor and mature sequences of miR-144 were obtained from miRBase^5^.

The GEO datasets series accession is GSE104441 (ID: 200104441). The GO map was analyzed by OmicShare Tools^6^.

Cis-element Cluster Finder^7^, Methprimer^8^, and JASPAR^9^ were used for promoter and transcription factors prediction.

### Statistical Analysis

Statistical analysis was carried out with the SPSS software (21.0 version, IBM, United States). All data were presented as means ± standard error (SE). Two-tailed Student’s *t*-test was used to compare average difference between the groups. *p* < 0.05 was regarded as a statistically significant difference.

## Results

### MiR-144 Promotes Pre-adipocytes Differentiation

As T2D is closely related to adipogenesis, and miR-144 is significantly upregulated in T2D, the role it plays still needs to be further investigated. To macroscopically ascertain the role of miR-144 in regulating adipogenesis, we screened the published GEO data.

**FIGURE 1:**
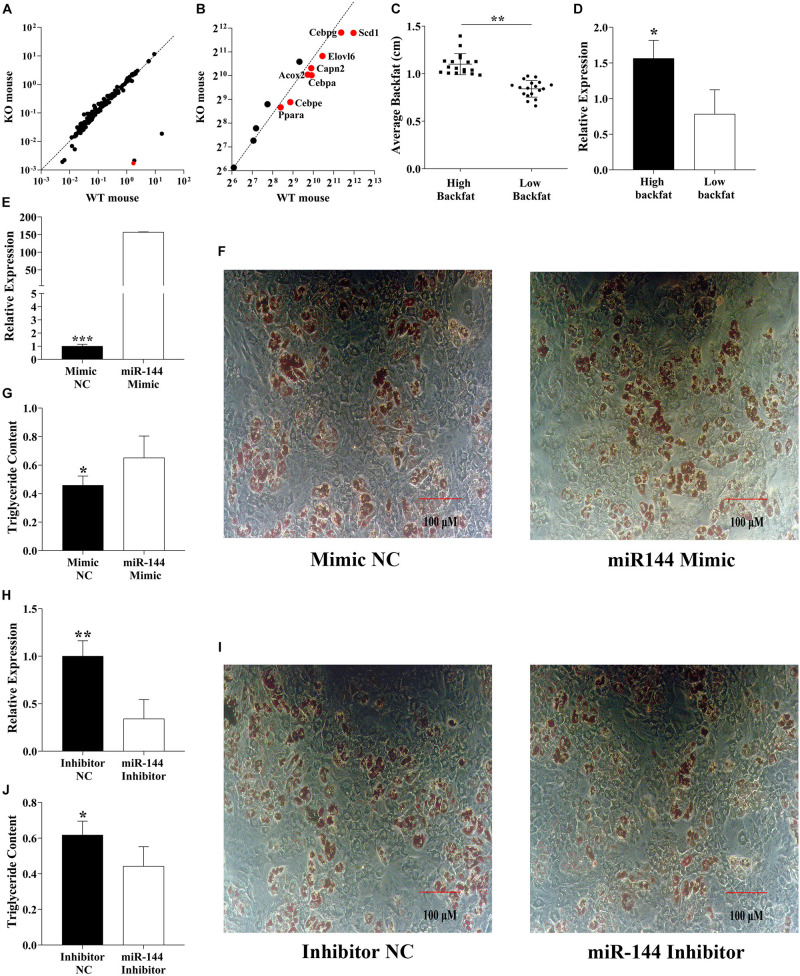
MiR-144 promotes pre-adipocyte differentiation. **(A)** Mouse knockout data from the GEO dataset (GSE104441) show the miR-144 specific knockout in mouse liver tissue. The red dot means miR-144. **(B)** Analysis of the adipogenic label genes in the GEO datasets (GSE104441). The above two dotted lines mean the standard line. **(C,D)** Detected miR-144 expressed in two backfat thickness groups. **(E–G)** Overexpressed miR-144-3p mimics in pre-adipocytes **(E)** then after 8-day differentiation inducer medium (DIM) stimulation, oil red O staining **(F)**, and triglyceride assay **(G)**, the results identified that miR-144 promoted pre-adipocyte differentiation. **(H–J)** Specific interference of miR-144 by its inhibitor confirms its regulatory function on adipogenesis. After statistical analysis, * in the figure indicates *p* < 0.05, **p < 0.01, and ***p < 0.001.

We found a miR-144 knock-out GEO dataset (GSE104441) (Figure 1A). By analysis, we found that some genes were involved in adipogenesis, mainly *C/EBP*α, *C/EBP*ε, *C/EBP*γ, *PPAR*α, *CAPN2*, and fatty acid synthesis-related genes, including *ELOVL6* and *SCD1* which were significantly downregulated (*p* < 0.05), as shown in Figure 1B.

However, these data were sequenced from the liver rather than adipose tissue, so we detected the miR-144 expression level in porcine white adipose tissue (WAT). The samples were divided into two groups (every group had 18 individuals) by the thickness of backfat (0.84 and 1.10 cm, respectively, *p* < 0.01), miR-144 in the high backfat group had an abundant expression level (*p* < 0.05), as shown in Figures 1C,D.

To confirm the data we detected, we transfected miR-144 mimics and inhibitors to porcine pre-adipocytes, as shown in Figures 1E,H, respectively. After the transfection, the samples were stimulated with DIM for 8 days, then we analyzed the adipogenesis of the pre-adipocytes. The results show that miR-144 mimics promoted pre-adipocyte differentiation, and miR-144 inhibitors suppressed the differentiation, as shown in Figures 1F,G,I,J, respectively.

This result further proved that miR-144 promotes pre-adipocyte differentiation, which is consistent with another paper (Shen et al., 2018).

### MiR-144 Targets 3′UTR of *FoxO1*

**FIGURE 2:**
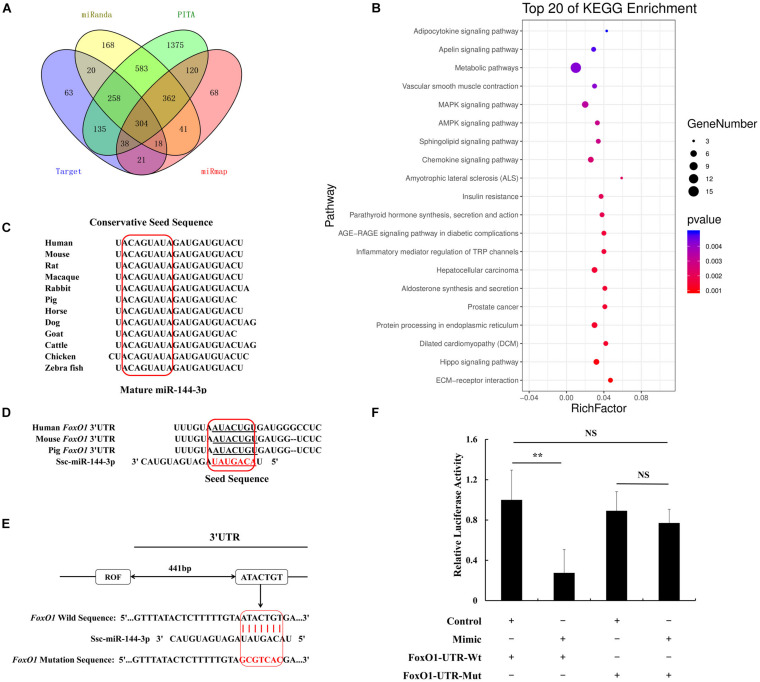
MiR-144 targets FoxO1 by binding in its 3′UTR. **(A)** The Venn diagram of target genes prediction data. **(B)** Top 20 KEGG enrichment that predicted genes by Venn diagram. Dot means genes number, and the color means *P*-value. **(C)** Conservation estimation of mature miR-144 sequences within different species. **(D)** Conservation estimation of miRNA response elements (MREs) between human, mouse, and pig. **(E)** The location of miR-144 MREs in porcine *FoxO1* gene 3′UTR. **(F)** The results of dual fluorescence activity detection of miR-144 targeting FoxO1. **p < 0.01.

miRNA can bind the miRNA response elements (MREs) located at 3′UTR of target genes to play a post-transcriptional role. We chose four different frequently used prediction software, including TargetScan, PITA, miRanda, and miRmap to predict the target genes of miR-144. In total, 304 potential targets were retrieved (Figure 2A). We used the GO analysis to study whether the above genes were involved in the adipogenic signaling pathway, results show there was an insulin pathway among the top 20 pathways. Insulin resistance characterizes T2D which in turn identifies miR-144 as a biomarker of T2D (Lettieri-Barbato and Aquilano, 2020). Here, the insulin pathway by GO analysis, respectively, contained four genes, phosphatase and tensin homolog (*PTEN*), forkhead box O1 (*FoxO1*), protein kinase C epsilon (*PRKCE*), and protein phosphatase 1 catalytic subunit gamma (*PPP1CC*) (Figure 2B).

Among them, FoxO1 has been demonstrated to be related to adipogenesis. Hence, FoxO1 was selected for this study. We found that mature miR-144 was conservative in the mass of species, as shown in Figure 2C. We estimated that the miR-144 MRE in *FoxO1 would be* located at 442–448 bp of its 3′UTR. The MRE in *FoxO1* was found to be shared between humans, mice, and pigs, which was conservative as well (Figures 2D,E). We respectively, constructed a recombinant pmirGLO plasmid containing the wild and mutational MRE of *FoxO1*. According to the luciferase reporter assay data, we proved that miR-144 targeted *FoxO1* (Figure 2F). The western blotting results by, respectively, transfecting miR-144 mimics and inhibitors also identified that miR-144 targeted FoxO1 (Figure 5H).

### FoxO1 Inhibits the Pre-adipocytes Differentiation

**FIGURE 3:**
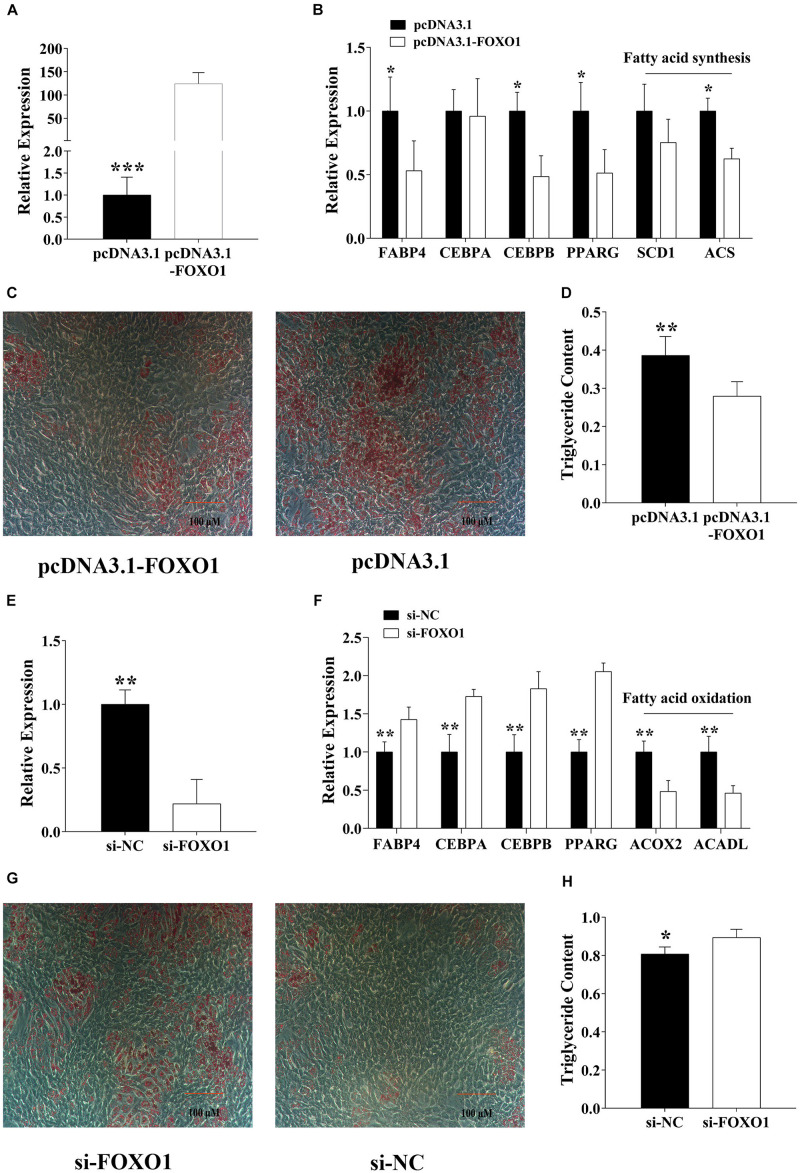
FoxO1 suppresses pre-adipocyte differentiation. **(A)** Overexpressed FoxO1 in pre-adipocytes. **(B)** Detected adipogenic label genes in pre-adipocytes after FoxO1 overexpression. **(C,D)** After 8-day differentiation inducer medium (DIM) stimulation, oil red O staining **(C)**, and triglyceride assay **(D)**. **(E)** Specific interference of FoxO1 by siRNA in pre-adipocytes. **(F)** After interference of FoxO1, the expression level of adipogenesis label genes in pre-adipocytes was detected. **(G,H)** After 8-day differentiation inducer medium (DIM) stimulation, oil red O staining **(G)**, and triglyceride assay **(H)**. *p < 0.05, **p < 0.01, and ***p < 0.001.

To identify the adipogenic role of *FoxO1*, we constructed a recombinant pcDNA3.1-FoxO1-CDs plasmid and transfected it to the pre-adipocytes by Lipofectamine 3000 for 48 h, then after 8-day DIM stimulation, we analyzed the adipogenesis (Figure 3A). Results show that, after FoxO1 transfection, the adipogenesis label genes, containing *FABP4*, *C/EBP*β, and *PPAR*γ *were* significantly downregulated, and fatty acid synthesis genes, for instance, *SCD1* and *ACS* were also significantly downregulated (*p* < 0.05, Figure 3B). Oil red O staining and a triglyceride assay further identified that FoxO1 inhibited pre-adipocyte differentiation (Figures 3C,D). On the other hand, we also found that si-FoxO1 regulated pre-adipocytes (Figure 3E). In the detection of the expression level of the label genes, we found that all of them were upregulated (*p* < 0.05), and that FoxO1 was involved in fatty acid oxidation. Thus we detected that *ACOX2* and *ACADL*, which were important label genes for fatty acid oxidation, were downregulated (*p* < 0.01).

As mentioned above, we believe that FoxO1 suppressed pre-adipocyte differentiation by regulating fatty acid synthesis and oxidation.

### MiR-144 and FoxO1 Co-regulate Adipogenesis

**FIGURE 4:**
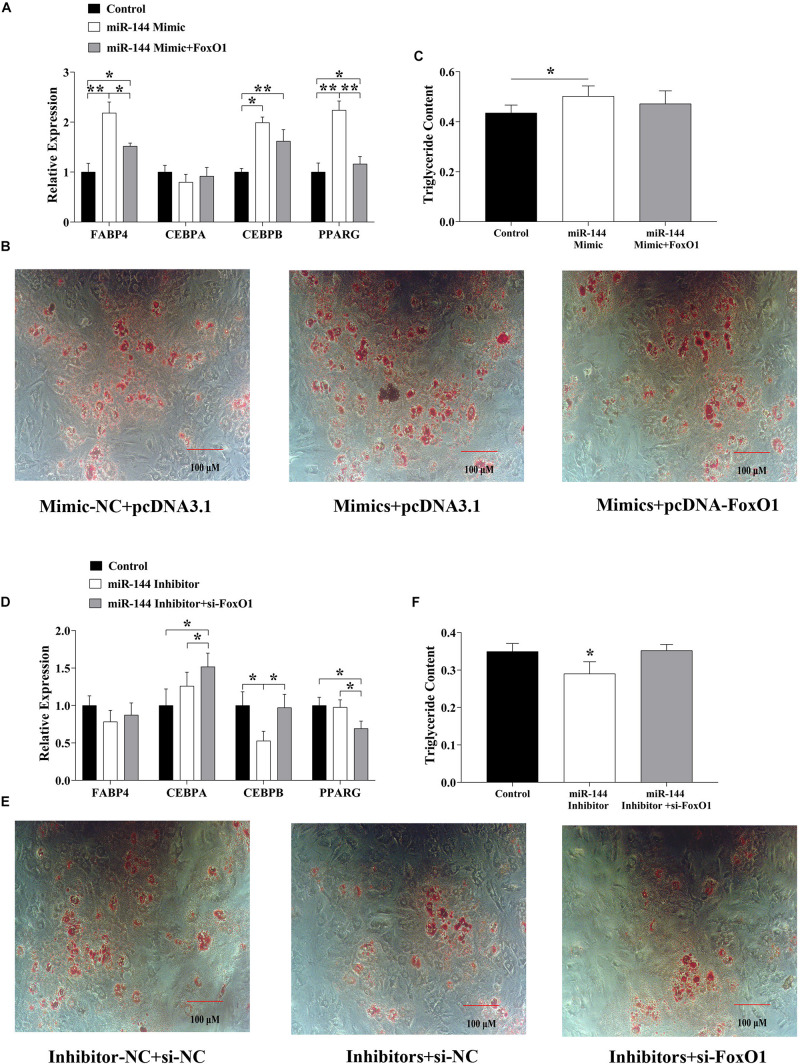
MiR-144 and FoxO1 co-regulated the adipogenesis. **(A)** Detected adipogenic label genes expressed in the miR-144 mimics and FoxO1 co-transfected group. **(B,C)** After 8-day differentiation inducer medium (DIM) stimulation, oil red O staining **(B)**, and triglyceride assay **(C)**. **(D)** Detected adipogenic label genes expressed in the miR-144 inhibitors and si-FoxO1 co-transfected group. **(E,F)** After 8-day differentiation inducer medium (DIM) stimulation, oil red O staining **(E)**, and triglyceride assay **(F)**. * *p* < 0.05, **p < 0.01.

The above results have demonstrated that miR-144 promoted adipogenesis while FoxO1 inhibited it, meanwhile miR-144 targeted FoxO1. But how they both co-regulate adipogenesis still needs to be further explored. Hence, we grouped miR-144 mimics and FoxO1 inhibitors, and si-FoxO1 to identify their regulation by co-transfecting pre-adipocytes. As the results show, when we co-transfected miR-144 mimics and FoxO1, some adipogenic label genes, for instance, *FABP4*, *C/EBP*β, and *PPAR*γ were upregulated in the co-transfected group compared with the control group, while *FABP4* and *PPAR*γ were downregulated compared with the mimic group (*p* < 0.05), as shown in Figure 4A. Further adipogenic-induced results show that there was no significant difference between the control and the co-transfected group while it was significantly less than the mimic groups (Figures 4B,C). On the other side, when we co-transfected miR-144 inhibitors and si-FoxO1, the results identified that *C/EBP*α and *C/EBP*β expressed in the co-transfected group was significantly more upregulated than the inhibitors group (*p* < 0.05), as shown in Figure 4D. Meanwhile, adipogenic-induced results also demonstrated that the co-transfected group recovered from the suppressed effect of adipogenesis found in the inhibitor group (Figures 4E,F).

**FIGURE 5:**
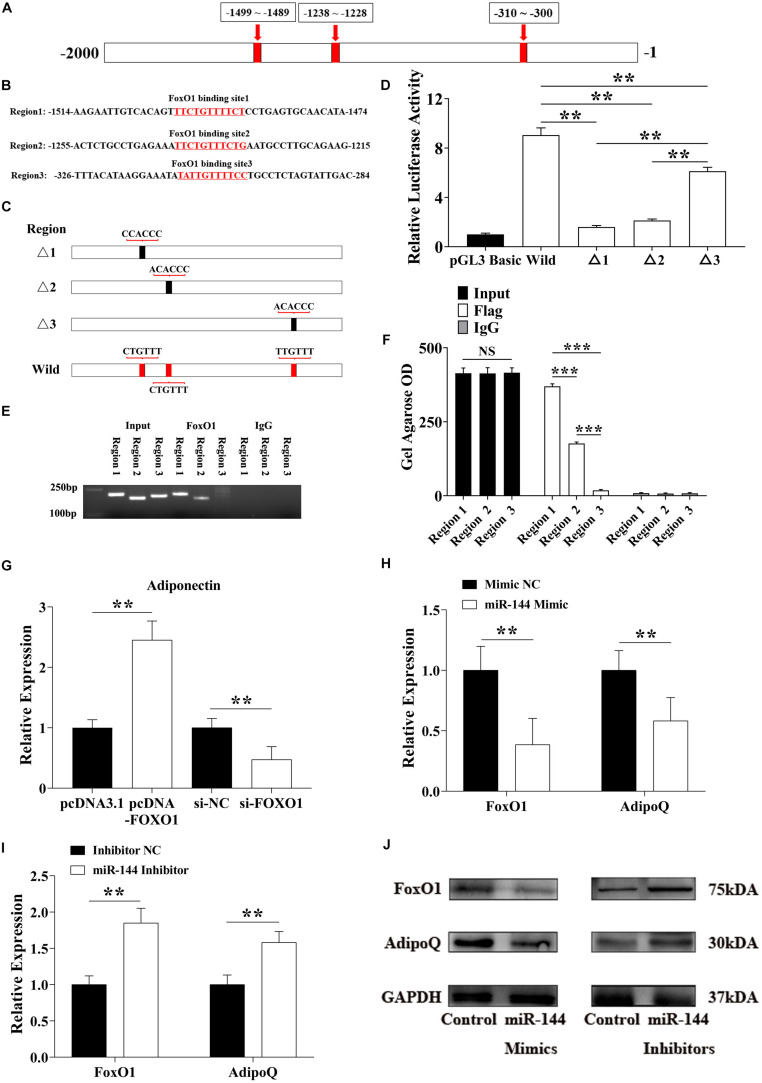
miR-144 inhibits FoxO1 to reduce its regulation of adiponectin and promote adipogenesis. **(A,B)** The specific binding location of the FoxO1 binding site in the -2,000 bp region **(A)** and the response element at each site **(B)**. **(C)** Constructed homologous recombinant pGL3 basic plasmid, including three mutational FoxO1 binding sites and a whole region contained three wild binding sites. **(D)** pGL3-regions directed luciferase gene reporter plasmid (pGL3-regions) was co-transfected with *FoxO1* encoding pcDNA3.1^+^ plasmids in porcine pre-adipocytes. Luciferase activity was measured 48 h after transfection. **(E,F)** ChIP-PCR detected the FoxO1 binding sites in the AdipoQ promoter region **(E)**, and the OD value was visualized by the ImageJ software **(F)**. **(G)** Detected adiponectin expressed level after FoxO1 overexpression and interference. **(H,I)** Detected FoxO1 and adiponectin expressed level after miR-144 mimics **(H)** and inhibitors **(I)** overexpressed in pre-adipocytes. **(J)** Western blotting analysis of FoxO1 and adiponectin proteins expression level after miR-144 mimics and inhibitors overexpressed in pre-adipocytes. **p < 0.01 and ***p < 0.001, respectively.

### MiR-144 Inhibits FoxO1 to Reduce Its Regulation of Adiponectin and Promote Adipogenesis

Adipose not only acts as an energy storage unit, but also as an important secretor. Adipose secrets a series of adipocytokines, among them, adiponectin is one of the most important. Adiponectin is the only adipocytokine to be found to play a negative regulatory role in adipogenesis so far.

In one report, FoxO1 bound the promoter of the *AdipoQ* gene in mice to activate the insulin signal pathway to promote fatty acid oxidation (Qiao and Shao, 2006). We chose the -2,000 bp region of the porcine *AdipoQ* gene transcriptional start site (TSS), and found three FoxO1 binding sites in this region (Figures 5A,B). To identify the most efficient promoter in the FoxO1 binding site, we constructed a homologous recombinant pGL3 basic plasmid, and found three mutational FoxO1 binding sites and a whole region containing three wild binding sites (Figure 5C). The co-transfection results revealed that binding site 1 and site 2 were, respectively, located at the −1,499 to −1,489 bp and −1,238 to −1,228 bp promoter regions. Both sites had high promoting efficiency, especial binding site 1 (Figure 5D). ChIP results further proved that only binding site 1 and site 2 had a PCR amplified band, the Gel Agarose OD showed that binding site 1 had significantly higher promoting efficiency than site 2 and site 3 (*p* < 0.01) (Figures 5E,F).

To confirm the results we detected, FoxO1 and si-FoxO1 were transfected to porcine pre-adipocytes, respectively. After 8-day DIM stimulation, endogenous adiponectin was detected, these results reconfirmed that FoxO1 promoted adiponectin expression by binding in its promoter region to regulate adipogenesis (Figure 5G). Moreover, as shown above, miR-144 inhibited FoxO1, here we, respectively, transfected miR-144 mimics and inhibitors to identify whether miR-144 affected adiponectin by inhibiting the FoxO1 expressed, and the results confirmed it (Figures 5H,I). Western blotting results further identified the above results (Figure 5J).

## Discussion

Adipogenesis has attracted great attention in the field of human health. Extreme adipogenesis leads to an increase in weight and even obesity which seriously endangers the health of mankind. Obesity is the source of most metabolic diseases, including hypertension, cardiovascular disease, T2D, and so on (Kopelman, 2000; Goran et al., 2003). In one report, approximately 40% of the population of the world suffer the risk of obesity, among them, nearly 20% are teenagers (Reinehr, 2018). So further adipogenesis molecular mechanism exploration is essential to resolve various diseases that threaten human health caused by obesity. However, in livestock production, the focus of research on adipogenesis differs slightly from the medicine field. For a better taste, muscles with a certain amount of intramuscular fat (IMF) are often pursued, which differs completely from the field of human health, after all, IMF is one of the prevalent causes of T2D. Though the focus is different between the above fields, the inquiry into the mechanism is consistent between them.

With an adipogenesis consensus developed, it has been gradually verified that in addition to the classical molecular mechanism that regulates adipogenesis, the role that epigenetics play is also becoming increasingly recognized. The role of epigenetics includes but is not limited to DNA methylation, histone modification, chromatin remodeling, maternal effect, and non-coding RNA (ncRNA). Among them, the mechanisms of DNA methylation and ncRNAs have been more thoroughly studied. In general, DNA methylation inhibits the transcription of genes, leading to a reduction in expression level. While ncRNA involves long non-coding RNAs (lncRNAs), circle RNAs (circRNA), pseudogenes, and miRNA. Except for lncRNAs, miRNAs are generally known as the most high-profile ncRNAs.

It is well known that miRNAs are involved in a mass of biological activities including adipogenesis, among them, some famous miRNAs including miR-27 and miR-130, have been demonstrated to play an important role in adipogenesis (Eun Kyung et al., 2011; Pan et al., 2014; Zhang et al., 2014). In this study, we found that miR-144 was significantly upregulated in WAT (*p* < 0.01) and we identified that it promoted pre-adipocyte differentiation (Figure 1). This result is consistent with the conclusion in another study that miR-144 promotes adipogenesis (Shen et al., 2018). The function of miRNAs mainly depends on binding in the 3′UTR of its target genes in order to play a post-transcriptional role, we proved that FoxO1 was one of its targets (Figure 2).

The FoxOs family contains four members, FoxO1, FoxO3, FoxO4, and FoxO6, which are nearly expressed in all tissues. Furthermore, the FoxOs oversee a mass of cellular processes, for instance, differentiation, proliferation, metabolism, apoptosis, autophagy, and stress resistance in numerous tissues. FoxO1 was the first to be discovered in humans, meanwhile in homozygous knockout mice it led to embryonic lethality (Hosaka et al., 2004). FoxO1 plays a crucial role in ruling the cell cycle and cellular metabolism (Li et al., 2017). Above all, FoxO1 is massively expressed in adipose tissue, where it is involved in regulating adipocyte differentiation and trans-differentiation, oxidative stress defense, and lipid metabolism (Gómezlópez et al., 2007; Lettieri Barbato et al., 2014). FoxO1 regulates the process of adipogenesis, it achieves this by acting as a regulator in insulin signaling. In detail, fasting stimulates the activation of the lipolytic pathway and promotes the breakdown of triglycerides and the releases of free fatty acids from adipose tissue, FoxO1, in this context, transcribes genes involved in lipid catabolism by signaling pathway inhibition, whereas, in the feeding condition the reverse is seen (Assimacopoulos-Jeannet et al., 1995; Lettieri Barbato et al., 2014; Ioannilli et al., 2020).

We subsequently detected the regulatory effect of FoxO1 on adipogenesis. Consistent with our findings, FoxO1 has a significant inhibitory effect on adipogenesis (Figure 3). To confirm the co-regulation of miR-144 and FoxO1 on adipogenesis, we co-transfected miR-144 and FoxO1. The results further proved that miR-144 and FoxO1 adipogenesis regulation is unified (Figure 4). However, there was another paper that demonstrated that miR-144 targeted *C/EBP*α. As one of the most crucial regulators of adipogenesis, C/EBPα is recognized as a positive factor of adipogenesis, thus the function of miR-144 on adipogenesis seems to be debatable (Itziar et al., 2017). However, our results clearly show that miR-144 has a promoting effect on adipogenesis. Perhaps there are mechanisms that need to be further explored.

FoxO1 is involved in the insulin signaling pathway as a regulator. FoxO1 can bind to a series of targets, we can finally verify that adiponectin is its target as confirmed by our estimation. Adiponectin is widely recognized as an inhibitor of adipogenesis, therefore, we only needed to detect whether FoxO1 had the ability to bind to the adiponectin gene promoter region and facilitate its expression. Moreover, miR-144 inhibits FoxO1, hence miR-144 would regulate adiponectin by inhibiting FoxO1. The results demonstrated that these two have a unified regulatory effect on adipogenesis, which confirms the hypothesis. Meanwhile, we further determined the key binding site of FoxO1 in the adiponectin gene promoter region, the results identified that binding site 1 and site 2 have significant promoting efficiency, especially site 1, which provides a basis for subsequent research (Figure 5).

In addition, regarding the regulatory function of miRNA, one of the most interesting hypotheses at present is the competitive endogenous RNAs (ceRNAs) hypothesis (Cesana et al., 2011). The theoretical basis of this hypothesis is based on the shared MREs between different target genes. In this study, we did not consider the ceRNA effect, the main reason is we believe that the “competitiveness” caused by a single miRNA is very limited, and the regulatory role of the overall endogenous environment has yet to be considered (Denzler et al., 2014; Chiu et al., 2018). However, if there is a co-interaction net containing more than one miRNA, the ceRNA effect is a regulatory perspective that is worth exploring.

## Conclusion

**FIGURE 6:**
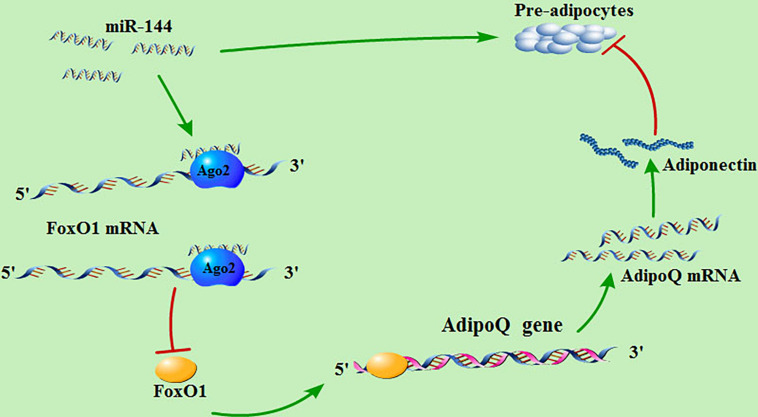
MiR-144 regulates the signaling pathway of adipogenesis in pre-adipocytes. miR-144 targets FoxO1, thus reducing its expression and inhibiting its promotional effect on adiponectin, thereby alleviating the inhibitory effect of adiponectin on adipogenesis.

As mentioned above, our research indicates that miR-144 targets FoxO1, thus reducing its expression and inhibiting its promotional effect on adiponectin, thereby alleviating the inhibitory effect of adiponectin on adipogenesis (Figure 6).

## Data Availability Statement

The datasets generated for this study can be found in the online repositories. The names of the repository/repositories and accession number(s) can be found in the article/ Supplementary Material.

## Ethics Statement

The animal study was reviewed and approved by the Animal Ethics Committee of the Nanjing Agricultural University.

## Author Contributions

WL conceived the idea, performed the analyses, and wrote the manuscript. WL and YT implemented the package. YZ and JZ contributed to the data analysis and checked the manuscript. WW and LZ checked the finished manuscript. JC conceived the idea, supervised the project analysis, and contributed to the manuscript preparation. All authors contributed to the article and approved the submitted version.

## Conflict of Interest

The authors declare that the research was conducted in the absence of any commercial or financial relationships that could be construed as a potential conflict of interest.

## Footnotes

1 http://www.mirbase.org/

2 http://www.pictar.mdc-berlin.de/

3 https://mirmap.ezlab.org/

4 http://cbio.mskcc.org/microrna_data/miRanda-aug2010.tar.gz

5 http://www.mirbase.org/

6 https://www.omicshare.com/tools/Home/Soft/seniorbubble

7 https://zlab.bu.edu/∼mfrith/cister.shtml

8 http://www.urogene.org/methprimer/

9 http://jaspar.genereg.net/
