# Supplementary material for: MiR-144-3p Targets FoxO1 to Reduce Its Regulation of Adiponectin and Promote Adipogenesis
Source: Front Genet. 2020 Dec 14;11:603144. doi: 10.3389/fgene.2020.603144 (PMC7767994; doi:10.3389/fgene.2020.603144)
Supplement: Supplementary file 1 [file Table_1.docx]

Supplementary Table1. The sequence used in the whole paper.

| Name | Sequence (5’-3’) | Length(bp) | TM/℃ |
| --- | --- | --- | --- |
| ACS qPCR | F:ACGGGACAAGGATGGCTATTA | 133 | 61 |
|  | R:CACGGCTGCCTCTGCGAT |  |  |
| ACOX2 qPCR | F:CTTTCTCCACGATGGCTTCC | 226 | 60 |
|  | R:GGCAGGGTTCCTCTGAGTATTG |  |  |
| AdipoQ qPCR | F:CGCCTATGTCTACCGTTCAGC | 196 | 60.5 |
|  | R:CTTCACATCCTTCAAGTAGACCGT |  |  |
| C/EBPα qPCR | F:CCTTCAACGACGAGTTCCTGG | 296 | 59.5 |
|  | R:CTGCTTCGACTCGTCCTCCTC |  |  |
| C/EBPβ qPCR | F:TAAGCACAGCGACGAGTACAAGA | 192 | 60 |
|  | R:TTGAACAAGTTCCGCAGGGT |  |  |
| FABP4 qPCR | F:GACAGGAAAGTCAAGAGCACCA | 229 | 60 |
|  | R:TCGGGACAATACATCCAACAGAG |  |  |
| FoxO1 qPCR | F:CTGTCCTACGCCGACCTCA | 255 | 60.5 |
|  | R:CCCACTCTTGCCTCCCTCT |  |  |
| GAPDH qPCR | F:GTTCCAGTATGATTCCACCCAC | 270 | 60 |
|  | R:TTCACGCCCATCACAAACAT |  |  |
| PPARγ qPCR | F:CAGAGTATGCCAAGAACATCCCT | 223 | 58.5 |
|  | R:GCAAACTCGAACTTGGGCTC |  |  |
| miR-144 qPCR | F:GGCCGGCTACAGTATAGATGATG | 67 | 60 |
|  | R:CGCAGGGTCCGAGGTATTC |  |  |
| U6 | F:CCTGACCATCGGTTCGCAA | 128 | 60 |
|  | R:CCCGGAACTACGCGGAGAA |  |  |
| AdipoQ Promoter | F:GGGGTACCATCACTGCCCATCTCATACC | 1434 | 60 |
|  | R:CCCTCGAGCCTCCTATAACAGGGACAGAA |  |  |
| AdipoQ ChIP 1 | TGGGGACAAACCATTGAACAG | 215 | 61.5 |
|  | GCTCAGTGAAGCGACTTACGAA |  |  |
| AdipoQ ChIP 2 | ACTGGGCTTTTCTGGACATACTTG | 187 | 60.5 |
|  | TCAGGACCCCAGCAACAACTT |  |  |
| AdipoQ ChIP 3 | TGGTTCCCTGGACTTATTAGAGC | 203 | 60.5 |
|  | GCCAAGTGGGGCTTCAGTC |  |  |
| FoxO1 CDs | F:GGGGTACCGGGTCACCATGGCCGAAGC | 1989 | 65 |
|  | R:CCCTCGAGAAAGGTGGGGTGAAGGACATC |  |  |
| FoxO1 UTR | F:CGAGCTCTTTCCTTTCGTCAGACTTGG | 469 | 60 |
|  | R:CCCTCGAGAGATTGGTAACAGGCTATTGG |  |  |
| FoxO1 siRNA | F:GCAUGUUCAUUGAGCGCUUTT |  | / |
|  | R:AAGCGCUCAAUGAACAUGCTT |  |  |
| miR-144 Mimics | F:UACAGUAUAGAUGAUGUAC |  | / |
|  | R:ACAUCAUCUAUACUGUAUU |  |  |
| miR-144 Inhibitor | F:GUACAUCAUCUAUACUGUA |  | / |

Note: AdipoQ Regions primer for pGL3-Basic, Kpn I for F primer, and Xho I for R primer. FoxO1 CDs primer for pcDNA3.1+, Kpn I for F primer, and Xho I for R primer. FoxO1 UTR primers for pmirGLO, Sac I for F primer, and Xho I for R primer. Construction of recombinant plasmid as followed:

FoxO1 CDs and 3’UTR region contained miR-144 binding site were cloned from porcine adipose tissue cDNA and AdipoQ promoter region was cloned by DNA, then recycled and purified the PCR products, to comfirm their completion by sequenceing. AdipoQ Regions primer for pGL3-Basic, Kpn I for F primer, and Xho I for R primer. FoxO1 CDs primer for pcDNA3.1+, Kpn I for F primer, and Xho I for R primer. FoxO1 UTR primers for pmirGLO, Sac I for F primer, and Xho I for R primer. Respectively enzyme cut FoxO1 CDs and pcDNA3.1+, FoxO1 3’UTR and pmirGLO, AdipoQ promoter and pGL3 basic plasmid for one hour at 37℃. T4 ligase connected plasimd and PCR products at 37℃ to be a constructed pcDNA3.1-FoxO1, pGLO-FoxO1-UTR and pGL3-AdipoQ. Primers used as shown in Table S1.
